# Effects of β-hydroxy-β-methylbutyrate (HMB) supplementation in addition to multicomponent exercise in adults older than 70 years living in nursing homes, a cluster randomized placebo-controlled trial: the HEAL study protocol

**DOI:** 10.1186/s12877-019-1200-5

**Published:** 2019-07-05

**Authors:** Javier Courel-Ibáñez, Jesús G. Pallarés, on behalf of the HEAL study group, Jesús G. Pallarés, Javier Courel Ibáñez, Ricardo Morán Navarro, Elena Saura Guillén, Alejandro Martínez Cava, Alejandro Sánchez Pay, Ángel Buendía Romero, Silverio García Conesa

**Affiliations:** Human Performance and Sports Science Laboratory, Faculty of Sport Sciences, University of Murcia, Calle Argentina, 19, 30720 San Javier, Murcia Spain

**Keywords:** Healthy ageing, Physical activity, Physical fitness, Falls, Dynapenia

## Abstract

**Background:**

Evidence supports the fact that multicomponent exercise and HMB supplementation are, separately, effective in improving older adult’s health and palliate functional metabolic diseases in older people. However, the true effect of HMB supplementation combined with a tailored exercise program in frail older adults is still unknown. Thus, the aim of the HEAL (HMB + Exercise = Adults Living longer) study is to assess the effects of the combination of a daily multicomponent exercise and resistance training (VIVIFRAIL program) intervention in addition to HMB supplementation on older adults’ health.

**Methods/design:**

A 24-week cluster randomized, double-blind, placebo-controlled study will be conducted on 104 adults ≥70 years. Nursing homes will be randomized to either of four groups: Ex-HMB (exercise intervention with HMB), Ex-Plac (exercise intervention with placebo), NoEx-HMB (no exercise intervention with HMB), and Controls (No exercise and no HMB). Intervention groups which include exercise will complete the individualized multicomponent (strength, balance and cardiovascular exercises) training program VIVIFRAIL. Intervention groups which include HMB supplementation will receive a 3 g/daily dose of free acid HMB in powder form. The primary outcome measure is the functional capacity. Secondary outcome measures are muscle strength and power, frailty and fall risk, body composition, biochemical analyses and cardiometabolic risk factor, disability and comorbidity, cognitive function and depression.

**Discussion:**

The findings of the HEAL study will help professionals from public health systems to identify cost-effective and innovative actions to improve older people’s health and quality of life, and endorse exercise practice in older adults and people living in nursing homes.

**Trial registration:**

NCT03827499; Date of registration: 01/02/2019.

## Background

To date, people are living more years than ever before in history and the world’s aging is rising at a staggering rate [1]. Thus, strategies focused on health maintenance for aging people through exercise and proper nutrition are required to contribute to lifelong wellbeing and prevent ageing diseases and chronic illness. Frailty, sarcopenia, dynapenia and sarcopenic obesity states are the main metabolic complications in older people and represent a major public health challenge [2–4]. A recent estimation from the Eurostat online database (28 European countries) suggests increments from 60 to 70% of individuals with sarcopenia in 2045 affecting 12.9 to 22.3% of people over 65 years old [5]. These diseases are caused by a degenerative loss of muscle mass (muscle wasting), strength and mobility. The combination of exercise with protein dietary supplementation is proven to be highly effective to increase muscle mass and strength in older adults [6–8].

Evidence states that exercise in older people is a main component in frailty prevention (increases strength and decreases falls incidence) and functional capacity preservation (increases mobility and autonomy) [8–10]. In turn, weak elders have greater risk of disability, hospitalization, morbidity and death [3, 11]. In addition to a better physical condition, exercise has a clear impact on psychological well-being in older people [12]. Although much remains to be done, the possibility of physical exercise as the new medication for the twenty-first century is truly inspiring. The first step of this revolution is that the question is turning from “may I prescribe physical activity for older people?” to “what kind of exercise must I prescribe?” [13]. Reducing sedentary behaviours and promoting exercise training in older adults living in nursing homes stands as a main global challenge [14, 15].

Very recently, the ERAMUS+ co-funding VIVIFRAIL project (http://www.vivifrail.com) has developed a multicomponent exercise program (strength, balance and cardiovascular exercises), carefully adapted, for improving functional capacity for older people above 70 years [16]. The program includes a practical guide for testing and prescribing the physical training according to each specific condition (serious, moderate, slight or no limitation, and with or without risk of falling). Furthermore, the VIVIFRAIL App allows individuals’ monitoring and provides clear instructions to effectively complete the program within the everyday environment. Now that long-term exercise interventions in older adults are more possible than ever [17], what is now required is to examine the effectiveness of this program on relevant health and functional outcomes for older adults and nursing homes residents [15].

The β-hydroxy-β-methylbutyrate (HMB) dietary is a bioactive metabolite formed from the decomposition of leucine, an essential branched-chain amino acid. The importance of leucine has anti-catabolic properties and plays an important role in protein metabolism, glucose homeostasis, insulin action and recovery from exercise [18–20]. A dose of 3 g of HMB dietary supplementation provides 60 g of leucine, which otherwise would imply 600 g of high biological value protein [21]. The HMB supplementation is affordable (around 50€/kg) and its consumption is safe with no adverse effects [22, 23]. In older adults > 60 years old, HMB is demonstrated to have anti-catabolic effect, enhance protein synthesis, attenuate proteolysis, increase muscle mass and decrease muscle damage [24–26]. Despite the fact that HMB efficacy varies [25], a meta-analysis concluded that HMB supplements contribute to the preservation of muscle mass in old age [27]. Based on these findings, the supplementation of HMB appears to be an effective strategy to prevent metabolic and physical complications in ageing (frailty, dynapenia, sarcopenia and sarcopenic obesity) and preserve health, functional capacity and strength in older people.

A recent investigation [28] reported for the first time a significant reduction of 50% in whole body plasma concentration of HMB and reductions of 25% on the conversion of leucine to HMB in older adults (~ 65 y-old). At the moment, there is limited understanding why this happens, but it seems clear than reductions in HMB conversion are associated with age [29]. These decrements on high quality protein synthesis importantly accounted for a decline in muscle weakness in older people [30]. Hence, the possibility of palliating muscle and functional losses in the ageing by HMB supplementation is truly inspiring and encourages further studies [26, 27].

To date, only five Randomized Control Trial (RCT) studies have examined the effectiveness of exercise training combined with HMB dietary supplementation in older adults > 60 years old [31–35]. Whereas it seems clear that HMB supplements contribute to the preservation of muscle mass in old age, contradictory evidence on its effects on strength increments [31–34] and functional performance [33–35] in older adults exists. These equivocal outcomes may be attributed to the protocols applied, with discrepancies in the training volume (number of sessions, time per session, number of reps), intensity (load monitoring and progression) and exercises. Besides, sample sizes explored are reduced (*n* < 32) and only one RCT [32] conducted an exercise intervention > 8 weeks. Consequently, there is a need for longer and larger studies to fully determine the potential effects of HMB supplementation on physical performance, translating to a functional benefit [34]. In this sense, the HEAL (HMB + Exercise = Adults Living longer) study will be the first RCT conducting a specific, individualized, multicomponent exercise intervention for the older adult population such as the VIVIFRAIL [16]. Because this evidence-based program has been proven as effective and safe in adults aged 65 years or over [17], it represents an excellent opportunity to determine the true effects of HMB supplementation in enhancing training performance. Furthermore, the promising results of HMB supplementation to mitigate age-related cognitive deficits [36, 37] and the lack of studies exploring its impact on people with very limited or no mobility [25, 38] encourage adopting this strategy in vulnerable people such as older nursing homes patients. Therefore, the aim of the HEAL study is to assess the effects of the combination of a daily multicomponent exercise and resistance training (VIVIFRAIL program) in addition to HMB supplementation on older adults’ health.

## Methods/design

### Study design and settings

**Fig. 1:**
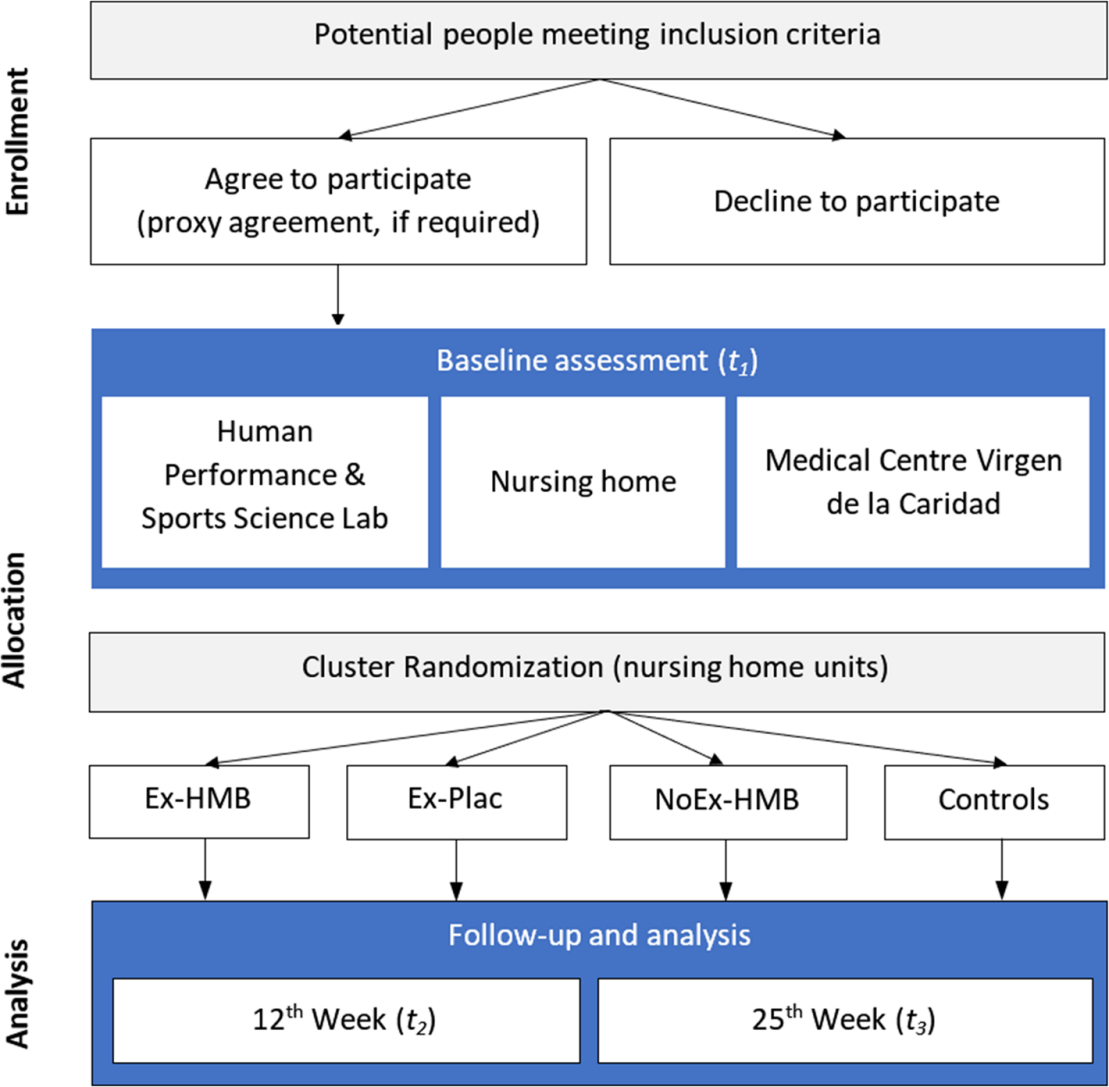
Flowchart of the trial

This is a cluster randomized, placebo-controlled study with four parallel groups. The study has been designed to determine the efficacy of HMB supplementation in addition to 24-weeks of multicomponent exercise and resistance training (the VIVIFRAIL program) in adults ≥70 years. Flowchart of the trial is shown in Fig. 1. Enrollment, intervention allocation, follow-up, and data analysis will be conducted according to the SPIRIT (Standard Protocol Items: Recommendations for Interventional Trials) statement [39, 40].

### Eligibility criteria

Inclusion criteria for enrollment will be: men and women aged ≥70 years, be able to follow an active physical rehabilitation program and voluntary participation. Enrollment of cognitively impaired older adults will require proxy permission (family member or caregiver) [41]. All potential participants will provide a medical history and undergo a medical examination to identify cardiovascular or metabolic conditions that would exclude participation (for full list, see Table 1).

**Table 1:** Exclusion criteria for the HEAL study

Exclusion criteria	
- Acute heart attack (recent 3–6 months) or unstable angina - Uncontrolled atrial or ventricular arrhythmias - Aortic dissecting aneurysm - Severe aortic stenosis - Acute endocarditis / pericarditis - Uncontrolled high blood pressure (> 180/100 mmHg) - Acute thromboembolism - Acute or severe heart failure - Acute or severe respiratory failure - Uncontrolled postural hypotension - Uncontrolled acute decompensated diabetes mellitus or low blood sugar - A recent fracture in the last month. - Coincident participation in any intervention trial - HMB contraindication, intolerance, or allergy - Have regularly performed exercise (> 20 min > 3 days/week) in the last 3 months - Malignant diseases (exceptions: basal or squamous-cell skin carcinoma or carcinoma in situ of the uterine cervix) - Revascularization within 1 year - Severe loss of vision, hearing, or communicative ability - Conditions preventing cooperation	

### Sample size

The required sample size will be determined on the basis on the functional capacity, using the Short Physical Performance Battery (SPPB) [42]. According to previous research on subjects with similar characteristics [17], a clinically relevant change is about 1.5 ± 1.0 points increments after 12-weeks. Differences of 2 point in total SPBB with a standard deviation of 3 points with a power of 80% and α of 0.05 can be estimated with 20 participants using the R software (v. 3.2.1) and the package *samplesize*. Assuming a maximum loss of follow-up of 30%, we will recruit 26 adults ≥70 years per group (*n* = 104). Similar interventions had an adherence rate of 75%, and a mean attendance of 80% to the sessions [17]. Therefore, the current estimation is realistic and affordable.

### Recruitment process and measurements procedures

A schematic overview of the outcomes, measures and timeline is shown in Table 2. Recruitment will be carried out in nursing homes in Murcia (Spain) located within a radius of 15 km or less than 20 min by car from the assessment sites. There are over 15 nursing homes among this radius that ensure recruitment of enough participants. Initial assessment will be carried out in the Human Performance & Sports Science Lab, Faculty of Sport Sciences, University of Murcia (Murcia, Spain). Body composition and biochemical analyses will be performed in the Medical Centre Virgen de la Caridad (Murcia, Spain). Participants will be scheduled in small groups to be taken to the laboratory for the initial assessment and medical centre. Dependent people will be transported in adapted vehicles with a caregiver. All measurements will be performed under technical and medical supervision.

**Table 2:** Schedule of enrollment, interventions, and assessments

TIMEPOINT	Enrollment -t_1_	Allocation 0	Baseline t_1_	Intervention	Follow-up 12th week post baseline t_2_	Close-out 25th week post baseline t_3_
ENROLLMENT						
Eligibility screen	✓					
Informed consent	✓					
Randomized Allocation		✓				
INTERVENTION				✓		
Ex-HMB				✓		
NoEx-HMB				✓		
Ex-Plac				✓		
Control				✓		
ASSESSMENTS						
*Functional capacity (primary outcome)*						
SPPB: Gait speed, balance, and 5-sit-to-stand	✓		✓		✓	✓
*Muscle strength and power*						
Grip strength			✓		✓	✓
1RM seated leg press			✓		✓	✓
1RM vertical bench press			✓		✓	✓
Sit-to-stand muscle power			✓		✓	✓
*Frailty and fall risk*						
Frailty phenotype			✓		✓	✓
Falls history	✓		✓		✓	✓
Fall risk assessment	✓		✓		✓	✓
*Body composition*			✓			✓
*Blood pressure and resting heart rate*			✓		✓	✓
*Haematology*			✓		✓	✓
*Biochemical analyses*			✓		✓	✓
*Nutritional status*			✓		✓	✓
*Sarcopenia*			✓		✓	✓
*Disability and comorbidity*						
Barthel index			✓		✓	✓
Lawton index			✓		✓	✓
Comorbidity			✓		✓	✓
*Cognitive function*	✓		✓		✓	✓
*Depression*			✓		✓	✓

Ex: 12-week of VIVIFRAIL multicomponent exercise program. HMB: dietary supplementation of HMB. *Plac* Placebo. *SPPB* Short Physical Performance Battery; *1RM* one-repetition maximum

The VIVIFRAIL exercise program will be administered by a training team (experienced and qualified personal trainers and physical therapists), under nursing supervision. After the initial assessment and one week before the start of the intervention, participants will attend a familiarization session at the place in which the testing and training will be conducted.

### Randomization and blinding

**Fig. 2:**
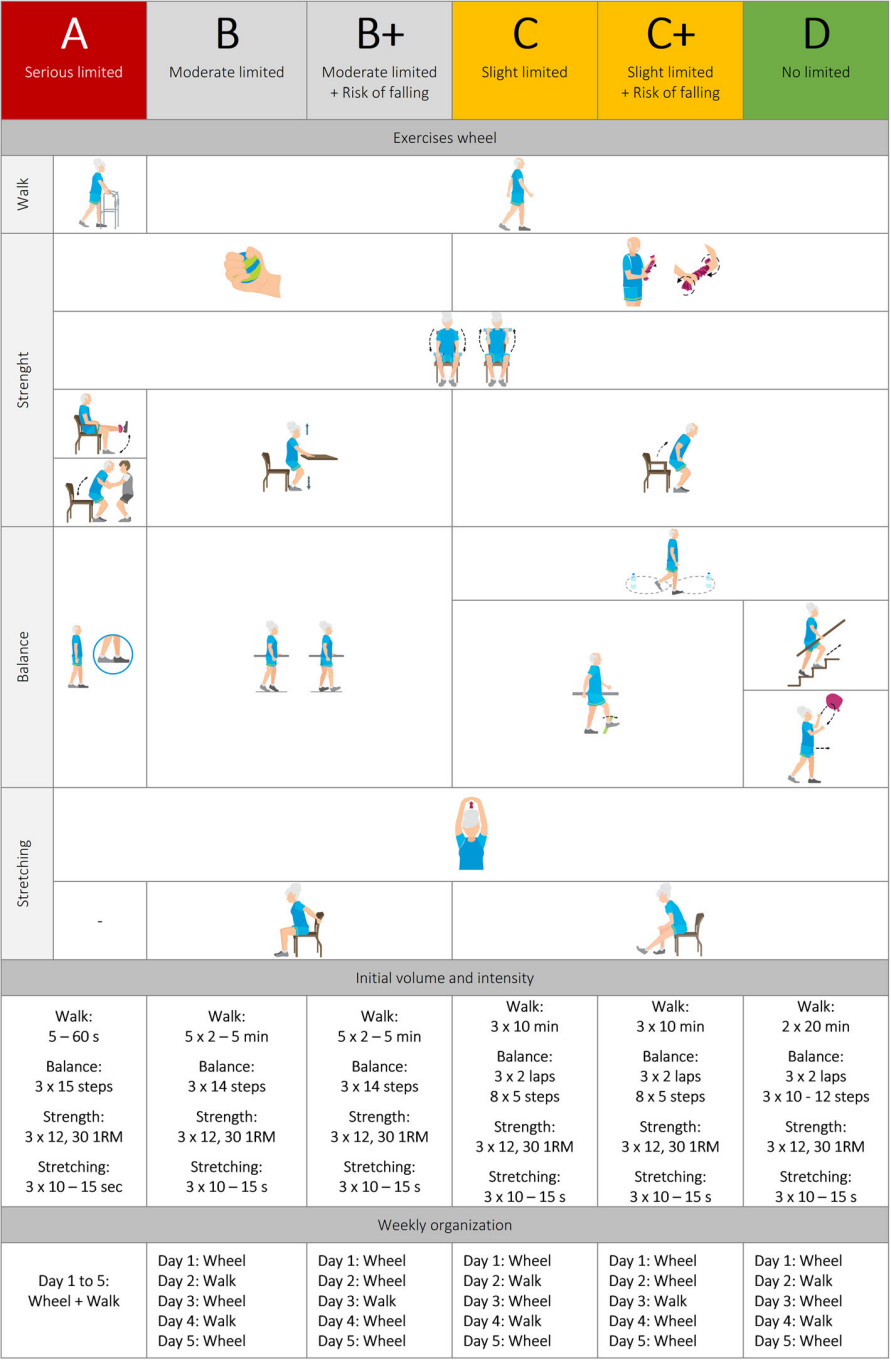
Summary of the VIVIFRAIL multicomponent exercise intervention program. Individualization based on baseline testing scores. Full guidelines: www.vivifrail.com

After recruitment and baseline measurements, nursing home will be randomized to either of the four groups in clusters, according to a computer-generated sequence using the Sealed Envelope Ltd. online system. The cluster design is chosen to prevent influences on participants’ behaviours within a given nursing home unit. A stratified randomization will be used according to their initial functional status (A, B, C or D, see Fig. 2) to reduce imbalance between groups. The allocation will be concealed in a password protected computer file. Given the nature of the treatment (i.e., daily exercise and dietary supplementation program), participants will be aware of their group allocation. Outcome assessors and data analysts will be blinded to the treatment group assignment. Assessors will not be involved in intervention activities. A dedicated protocol will be defined to protect the confidentiality of data.

### Interventions

#### Dietary supplementation

Intervention groups including HMB supplementation (Ex-HMB and NoEx-HMB) will receive a 3 g daily dose of free acid HMB in powder form (myprotein.co.uk, Cheadle, Cheshire, UK) dissolved freely into 250 mL of water during a 24-week intervention [34, 43]. Nursing staff will supply the doses as a part of their daily diet routine. Ex-Plac and Control groups will receive stevioside. Supplements will be packaged in indistinguishable envelopes and boxes, with an identification code for each participant and group. The compliance of supplementation will be monitored and ensured by medical staff working at the nursing home. Oral supplement and Vitamin D will be provided to maintain an acceptable nutritional status.

#### Multicomponent physical exercise program

Intervention groups including exercise (Ex-HMB and Ex-Plac) will complete an individualized multicomponent training program, VIVIFRAIL [16], 5 days a week during 24 weeks. Free on-line resources and program guidelines are available online (http://www.vivifrail.com/resources). The VIVIFRAIL program has been carefully designed for people ≥70 years and includes six programs or “Passports” adapted for each participant’s condition according to their functional limitation (serious [A], moderate [B], slight [C] and no limitation [D]) and risk of falling (B+ and C+). Individualization is made based on the baseline testing scores (i.e., the VIVIFRAIL test). Each program combines strength, power, balance, walking, stretching and cardiovascular exercises, in the named “VIVIFRAIL Wheel”. Training sessions are daily and weekly organized (type of exercise, steps and reps) in individual “Passports”. A summary of the exercise program is shown in Fig. 2.

The VIVIFRAIL program has a free mobile app available on iOS and Android, the latest version of which, (launched in January, 2019) allows the recording of the results of the test to automatically assign each participant to a training program. The App includes a calendar with a daily progression, training monitoring and rate of perceiving effort assessment.

### Outcome measures

*Functional capacity (primary outcome):* The Short Physical Performance Battery (SPPB) [42] scores from 1 (low mobility) and 12 (full mobility) points based on three tests: balance tests (tandem, semi-tandem and one foot next to the other), gait speed and 5-sit-to-stand test. The SPPB has been extensively administrated in older adults [17, 44]. The resulting scores are part of the VIVIFRAIL test to determine each individual’s physical exercise program [16].

*Maximal muscle strength and power output:* grip strength measurement (Jamar digital dynamometer, NexGen ergonomics, Pointe Claire, Quebec, Canada) [45], one repetition maximum (1RM) seated leg press and vertical bench press strength (Salter Ltd., Barcelona, Spain) and muscle power (T-Force Dynamic Measurement System, Ergotech Consulting SL, Murcia, Spain) [46, 47], sit-to-stand muscle power [48], in the same order with a 3-min rest between tests in order to diminish fatigue [49].

*Frailty and fall risk*: frailty phenotype determination [50], complete falls history and fall risk assessment, physical examination [16].

*Body composition:* Body composition will be assessed using dual-energy x-ray absorptiometry - DXA (Hologic, Bedford, MA; Discovery A), between 6:00 AM and 9:00 AM after a ≥ 10-h fast and after participants had voided their bladders [51].

*Blood pressure and resting heart rate:* Systolic and diastolic blood pressure, as well as resting heart rate will be measured after 10 min of rest, two times 2 min apart (M6 upper arm blood pressure monitor Omron. Omron Health Care Europe B.V. Hoofddorp, The Netherlands).

*Haematology*: Erythrocyte count, haematocrit, haemoglobin, platelets, leukocytes and erythrocyte mean corpuscular volume will be quantified by Coulter Cell Counter.

*Biochemical analyses*: glucose, high-density lipoprotein (HDL), total cholesterol (TC), triglycerides (TG), glycosylated haemoglobin (HbA_1c_), thyroid-stimulating hormone (TSH), C-reactive protein (CRP), albumin, prealbumine, transferrin, insulin-like growth factors (IGF-1 and IGFBP-3), creatine phosphokinase (CPK) and 25-hydroxyvitamin D (25[OH]D). Blood analysis will be conducted with standard methods using an autoanalyzer. Insulin sensitivity will be derived from the homeostatic model assessment for insulin resistance (HOMA-IR).

*Nutritional status:* The Mini-Nutritional Assessment (MNA-SF) [52] will be used to evaluate nutrition status and malnutrition risk.

*Sarcopenia*: The SARC-F will be used to diagnose sarcopenia [53].

*Disability and comorbidity:* Barthel index [54] and Lawton index [55] will be used to assess disability in basic activities and instrumental activities of daily living, respectively. Given the limitations of comorbidity indexes in older people [56], we will consider comorbidity when a participant presents two or more geriatric syndromes from a list of selected geriatric syndromes, as previously proposed [57].

*Cognitive function and depression:* the validated Spanish version of the Mini-Mental State Examination (MMSE) [58] will be used to assess cognitive function. Depression will be assess with the Spanish version of the 15-item Yeasavage geriatric depression scale [59].

### Statistical analysis

Analysis will be performed on participants who attended at least 80% the training sessions and completed all the measurements. Treatment effects will be tested using generalized linear models. All models will be adjusted for the baseline outcome value and repeated adjusting for gender, age, the group effect, and confounding factors.

### Trial registration

The trial was registered on ClinicalTrials.gov (identifier: NCT03827499) on 01/02/2019.

## Discussion

This paper outlines the protocol for a randomized, placebo-controlled study to determine the efficacy of HMB supplementation in addition to 24-weeks of multicomponent exercise and resistance training in adults ≥70 years old. At the time of writing, the study was ongoing (recruitment status). Baseline assessment is planned to started in March 2019.

Maintaining old people’s health and protecting them from frailty, muscle waist and cardiovascular diseases will save billions in public care costs by lengthening people’s healthy life, reducing the loss of income due to premature death and reducing nursing dependency [60]. Evidence supports that multicomponent exercise [8–10, 17, 61] and HMB supplementation [24–26] are effective in improving older adult’s health and palliating functional metabolic diseases in older people. However, the true effect of HMB supplementation combined with a tailored exercise program is still unknown. Just a few trials have investigated the combination of both [31–33], showing promising results. Moreover, the implementation of the new VIVIFRAIL multicomponent exercise program for frail old people in addition to HMB supplementation is still to be done.

The study results will be of high relevance to old people living in nursing homes and their health care providers. If the benefits of the combined VIVIFRAIL and HMB are proven, this could be an alternative management strategy to consider in nursing homes with older adults and people with functional metabolic diseases and muscle-wasting conditions. In addition, the current exercise intervention is inexpensive and freely available (http://www.vivifrail.com/), which permits its replication. The findings of the HEAL study will help professionals from public health systems to identify cost-effective and innovative actions to improve older people’s health and quality of life, and endorse exercise practice in older adults living in nursing homes.

## Data Availability

Data are not available due to EU General Data Protection Regulation. Please, contact the corresponding author if you are interested in study materials.

## Abbreviations

1RM: One-repetition maximum
CRP: C-reactive protein
DXA: Dual-energy x-ray absorptiometry
Ex-HMB: Exercise intervention with HMB
Ex-Plac: Exercise intervention with placebo
HDL: High-density lipoprotein
HMB: β-hydroxy-β-methylbutyrate
MMSE: Mini-Mental State Examination
NoEx-HMB: No exercise intervention with HMB
RCT: Randomized Control Trial
SPIRIT: Standard Protocol Items: Recommendations for Interventional Trials
SPPB: Short Physical Performance Battery
TC: Total cholesterol
TG: Triglycerides
TSH: Thyroid-stimulating hormone
